# MicroRNA expression and their molecular targets in food allergies: a systematic review

**DOI:** 10.3389/fimmu.2025.1524392

**Published:** 2025-05-12

**Authors:** Tekan Singh Rana, Rishipal Rastrapal Bansode, Jenny Pakhrin Rana, Leonard L. Williams

**Affiliations:** ^1^ Center for Excellence in Post-Harvest Technologies, North Carolina Agricultural and Technical State University, Kannapolis, NC, United States; ^2^ Department of Biology, North Carolina Agricultural and Technical State University, Greensboro, NC, United States

**Keywords:** food allergy, miRNA, RNAi, peanut allergy, CMA, BALB/c, NGS, ovalbumin

## Abstract

**Introduction:**

MicroRNAs (miRs) play an essential role in adaptive and innate immune systems by regulating the development of immune cells. However, detailed studies of miRs’ role in food allergies are scarce compared to those of other allergic or non- allergic diseases. This systematic review aims to study miRs expression and its role in food allergies (FAs) and determine the signature miRs in FAs.

**Method:**

Research articles published since 2015 were selected from various databases: Scopus, PubMed, ScienceDirect, and Web of Science. Randomized clinical trials, observational clinical studies, and *in vivo* studies were assessed via the Cochrane Risk of Bias 2 tool, the Newcastle-Ottawa scale, and SYRCLE method, respectively. The characteristics of the included studies, population characteristics, and experimental details were extracted, and the data were synthesized narratively.

**Result:**

MiRs expression had been investigated in the context of cow milk allergy (CMA) and peanut allergy (PA) through both *in vivo* studies and clinical trials. Clinical trials included allergies to multiple combined foods, individual foods (such as milk, peanut, and what), and drugs and venom, while *in vivo* studies were conducted on milk, egg, and peanut allergies. MiR-146a, miR- 155, and miR-30a-5p were common miRs between *in vivo* studies and clinical trials. Moreover, few miRs were commonly studied between different types of food allergies. In clinical trials, miR-143-3p was studied in peanut allergy and non-celiac wheat sensitivity (NCWS), and miR-155 was studied in CMA and egg allergy in *in vivo* studies. Furthermore, the same miRs varied on their molecular target and effect depending on the type of food allergy.

**Discussion:**

The study on signature miRs and their molecular target determination for the therapeutic purpose of food allergy is in its initial stage. For individual food allergies, miRs determination via next-generation sequencing (NGS), their validation via polymerase chain reaction (PCR), and target molecule determination via RNA interference (RNAi) should be the focus of future studies in order to determine reliable signature miRs of food allergy.

## 1 Introduction

**Figure 1:**
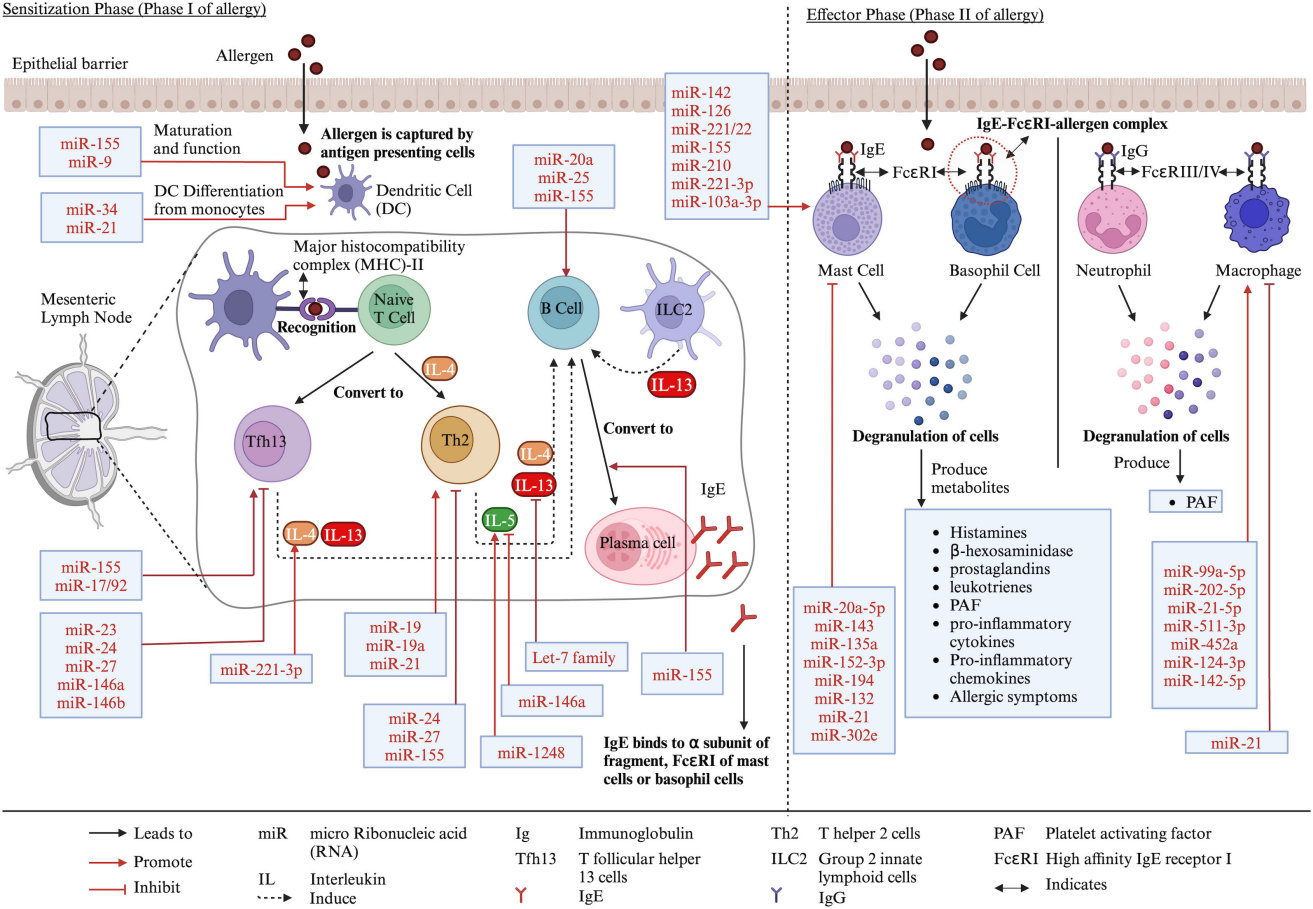
Different stages of food allergy development and role of miRs in the process. Created with BioRender.com.

Approximately 2%–4% of adults and 4%–6% of children are affected by food allergies worldwide (1). Food allergies are adverse reactions of the body to food proteins. It can be divided into two categories: immunoglobulin E (IgE)-mediated and non-IgE-mediated (2, 3). The IgE-mediated food allergies occur in two stages: sensitization and effector phases (3, 4). In the sensitization phase, food proteins enter the body through the epithelial barrier, and they are captured by antigen-presenting cells (APCs) such as dendritic cells (DCs) or macrophages. These APCs engulf and break the allergens into peptides and are presented by major histocompatibility complex class II (MHC-II), which is recognized by naive CD4^+^ T cells in mesenteric lymph node (MLN) (2, 4–6). The naive T cell then convert into T helper 2 (Th2) cells in the presence of interleukin-4 (IL-4) and produce IL-4, IL-5, and IL-13. These cytokines, in combination with the interaction of CD40 of B cells and CD40-ligand of Th2 cells, activate and convert IgG-producing B cells into IgE-producing plasma cells (3–6). Moreover, according to a current review, T follicular helper 13 (Tfh13) cells, which produce IL-4 and IL-13, and type 2 innate lymphoid cells (ILC2), which produce IL-13, help to expand Tfh and B cells, which further induce IgE production from B cells (3). The plasma cell-produced IgE, binds to the α subunit of fragment crystallizable epsilon region R1 (FcεRI) receptors of mast cells or basophil cells. The re-exposure of the allergen (called effector phase) to IgE-FcεRI causes cross-linking of the two FcεRI-bound IgE, making an IgE-FcεRI–allergen complex, which degranulates mast cells or basophil cells. The degranulation leads to the production of various mediators such as histamines, β-hexosaminidase, prostaglandins, leukotrienes, platelet-activating factors (PAFs), pro-inflammatory cytokines, and chemokines and the development of allergic symptoms (2–5). In neutrophils and macrophages, the activation occurs due to IgG-FcγRIII–allergen or IgG-FcγRIV–allergen complex-releasing PAFs (3) (**Figure 1**).

**Figure 2:**
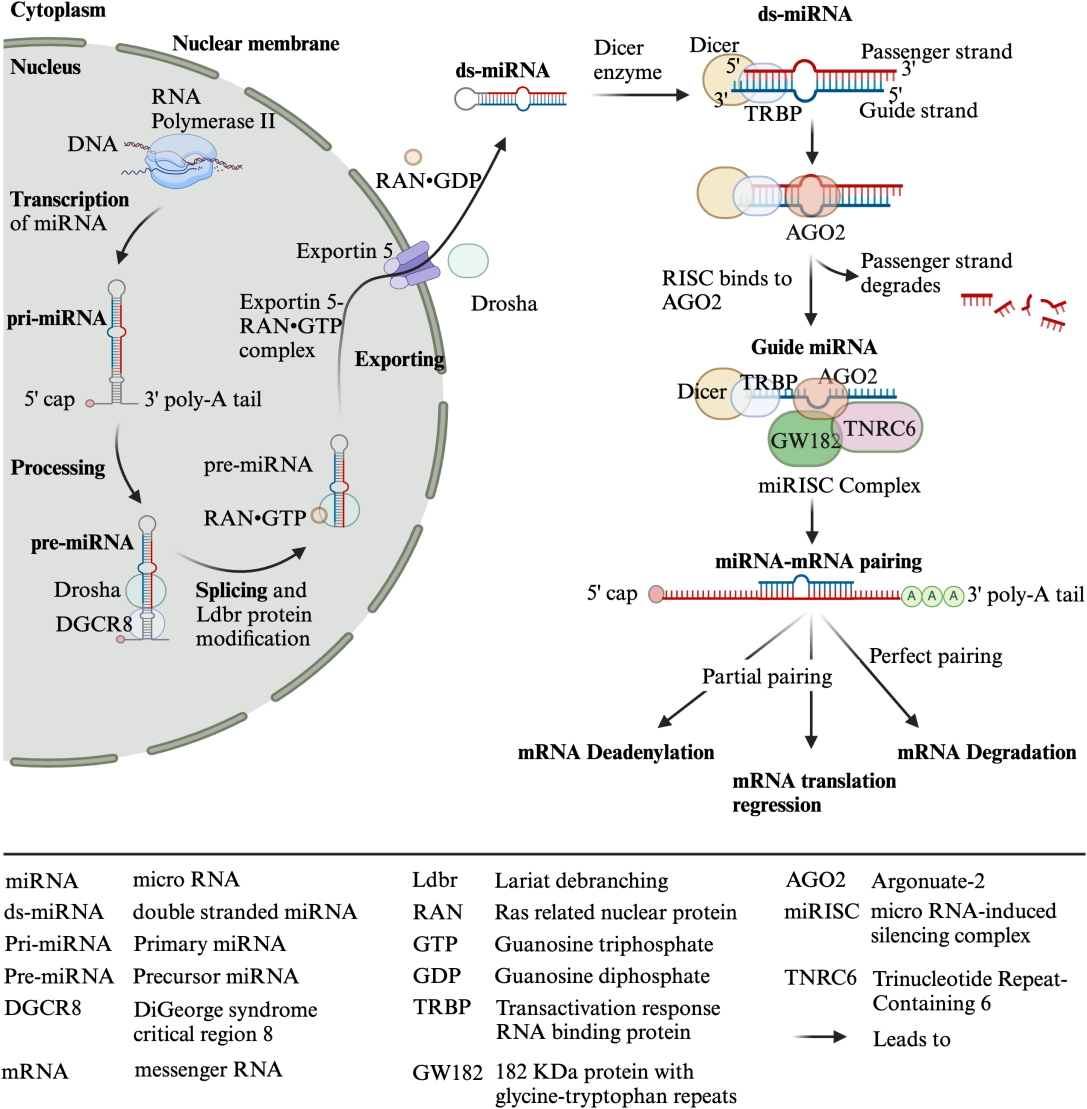
Biosynthesis of miRNAs. Created with BioRender.com.

MicroRNAs (miRs) are widely studied 19-22-nucleotide-long single-stranded non-coding RNAs that regulate gene expression post-transcriptionally (7). The miR-encoding genes are mainly located in the intron, and few are located in exons, intergenic regions, and 5′ and 3′ untranslated regions (UTRs). RNA polymerase-II transcribes miR-encoding genes, producing a single-stranded multiple hairpin structure protected by 5′-end caps and 3′-end poly-A tails, which are called primary miR transcripts (pri-miRs) (8–13). These pri-miRs are further processed by a class 2 RNase III endonuclease (Drosha) and its cofactor DiGeorge syndrome critical region 8 (DGCR8) in the nucleus into 65-nucleotide-long stem-loop-structured precursor miR (pre-miR). However, splicing of hairpin intron and modification with Lariat debranching (Ldbr) protein can also convert pri-miRs into pre-miRs (7, 10, 11, 13). Pre-miRs are exported from the nucleus to the cytoplasm by the ras-related nuclear protein (RanGTP)/exportin-5 complex. The exported pre-miRs are further processed (i.e., hairpin cut and convert pre-miR into -3p or -5p segments) by Dicer and transactivation response RNA-binding protein (TRBP) into 21 nucleotide long miR duplex with overhang, which are called double-stranded miRs (ds-miRs) intermediates. The ds-miRs unwind, and the guide strand of ds-miRs interacts with argonaute 2 (AGO2) protein, which results in functional miRs, while the passenger strand of the ds-miRs degrades. The functional miRs recruit components of the miR-induced silencing complex (miRISC), such as GW182, which interact with the AGO protein and bridge the AGO to other proteins involved in miRISC (e.g., 5′ de-capping deadenylase and exonuclease enzymes, which degrade target RNAs). Moreover, TRBP helps the binding of Dicer-miR with the RISC complex, which contains AGO2 and trinucleotide repeat-containing gene 6 protein (TNRC6) (7, 8, 10–12) (**Figure 2**).

Among four types of AGOs (AGO1–4), AGO2 is the most important for mammals, which has an active endonuclease pocket, binds to miRs, supports the DICER-dependent miR maturation process, and cleaves complementary RNA (10). MiRs bind on the 3′ or 5′ UTR or coding region of the target mRNAs, either degrading them (if completely paired with the target gene, which occurs occasionally in animals) or inhibiting their translation (if partially paired with the target) or promoting the translation of the target by binding to elements of the 5′ UTR (8–11, 13, 14) (**Figure 2**). Moreover, in the case of the complete pairing, AGO2 cleaves the target mRNA. However, in partial pairing, TNRC6 dominates the target mRNA’s deadenylation, degradation, or translation repression (7). In addition to direct binding to target genes, miRs also bind to RNA-binding proteins (RBPs), preventing them from binding and functioning as transcriptional activators or repressors (13).

MiRs are found in body fluids, reflecting the body’s physiological condition. Therefore, they have the potential to be used as signature biomarkers for various diseases (15). MiRs are essential in adaptive and innate immune systems, as they play important roles in the development, proliferation, activation, and inhibition of immune cells as well as cytokine production and their target genes, which are explained in detail somewhere else (9–11, 16–19). As an example, the role of miRs in immune cells related to allergy is described as follows. For T cells, miR-24, miR-27, and miR-155 reduced Th2 production, and miR-19, miR-19a, and miR-21 increased Th2 production (16, 19, 20). Moreover, miR-23, miR-24, miR-27, and miR-146a/b restricted Tfh cells, and miR-155 and miR-17/92 promoted Tfh cells (16). For DCs, miR-155 and miR-9 promoted DC maturation and function, and miR-34 and miR-21 promoted their differentiation from monocytes (16, 20). For B cells, miR-20a, miR-25, and miR-155 promoted B-cell responses (16). MiR-155 is also important for the differentiation of B cells into IgE-producing plasma cells (20). For macrophages, miR-99a-5p, miR-202-5p, miR-21-5p, miR-511-3p, miR-452a, miR-124-3p, and miR-142-5p promoted M2 macrophages, which are necessary for allergic reaction (17). However, miR-21 is also reported to negatively regulate macrophage activation (20). For mast cells, miR-142, miR-126, miR-221/22, miR-155, miR-210, miR-221-3p, and miR-103a-3p promote mast cell activation, leading to degranulation and cytokine release, while miR-20a-5p, miR-143, miR-135a, miR-152-3p, miR-194, miR-132, miR-21, and miR-302e decreased mast cell activation (21–23). For cytokines, miR-221-3p and miR-1248 upregulated IL-4 and IL-5, respectively, while miR-146a and let-7 family downregulated IL-5 and IL-13, respectively (19) (**Figure 1**).

It is noteworthy to mention that in addition to miRs, other epigenetic mechanisms such as histone modification and DNA methylation also affect allergies (24, 25). Moreover, these epigenetic mechanisms and miRs mutually regulate each other. For example, miRs can affect other epigenetic mechanisms by modulating enzymes important for histone modification and DNA methylation, such as DNA methyltransferases (DNMTs), histone deacetylases (HDACs), and polycomb-group genes. On the other hand, the epigenetic mechanisms could have transcriptional control on miRs expression (26–28). One of many reasons for miRs’ epigenetic regulation could be due to the fact that approximately 50% of miRs’ genes are located in CpG islands where DNA methylation occurs (27). Despite the interrelationship among epigenetics, miRs, and allergic disease/food allergy, that aspect is beyond the scope of this systematic review; thus, our current study only focuses on miRs expression in food allergy.

Moreover, the role of miRs in various types 2 immune diseases such as allergic rhinitis (29–31), atopic dermatitis (32–34), allergic asthma (7, 10), asthma (35–37), eosinophilic esophagitis (38), allergic contact dermatitis (39–41), and autoimmune diseases (11) have been studied and summarized elsewhere, and all agree that each miR has its distinct and critical role in type 2 immune system. However, compared to those of other diseases, studies on the effect of miRs on food allergy are scarce. Thus, this systematic review aims to study the signature miRs and their molecular targets in food allergies (FAs).

## 2 Materials and methods

### 2.1 Search strategy

**Figure 3:**
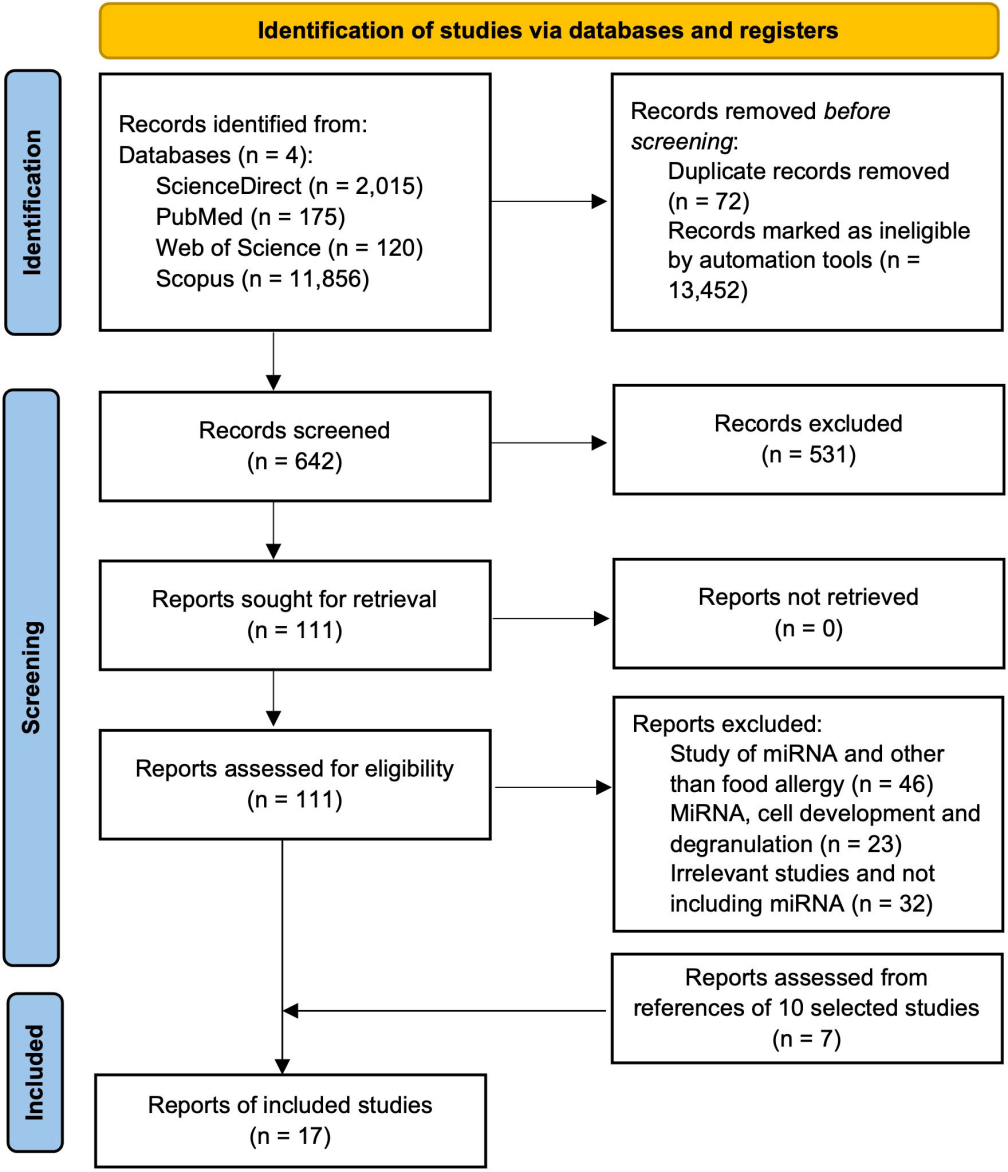
PRISMA flow diagram showing the steps of study selection process for the systematic review.

Articles published from 2015 to June 14, 2024, in four databases were searched: ScienceDirect, Web of Science, Scopus, and PubMed. Two authors (T.S.R. and J.P.R.) used the following combinations of keywords (three to four sets) in each database to get relevant articles: in ScienceDirect, a) (“food hypersensitivity” OR “food allergy” OR “allergy”) AND (“MicroRNAs” OR “miR”) AND (“proanthocyanidin” OR “anthocyanidin” OR “procyanidin” OR “flavonoids”), b) (“food hypersensitivity” OR “food allergy” OR “allergy”) AND (“MicroRNA” OR “miR”) AND (“mast cell” OR “basophil” OR “RBL-2H3”), and c) (“MicroRNAs” OR “miR”) AND (“mast cell” OR “basophil” OR “RBL-2H3”) AND (“proanthocyanidin” OR “anthocyanidin” OR “ procyanidin” OR “flavonoids”). In Web of Science, a) (“food hypersensitivit*” OR “food allerg*” OR “allerg*”) AND (“MicroRNA*” OR “miR*”) AND (“proanthocyanidin*” OR “anthocyanidin*” OR “procyanidin*” OR “flavonoid*”), b) (“food hypersensitivit*” OR “food allerg*” OR “allerg*”) AND (“MicroRNA*” OR “miR*”) AND (“mast cell*” OR “basophil*” OR “RBL-2H3*”), and c) (“MicroRNA*” OR “miR*”) AND (“mast cell*” OR “basophil*” OR “RBL-2H3*”) AND (“proanthocyanidin*” OR “anthocyanidin*” OR “ procyanidin*” OR “flavonoid*”). In Scopus, a) (“food hypersensitivit*” OR “food allerg*” OR “allerg*”) AND (“MicroRNA*” OR “miR*”) AND (“proanthocyanidin*” OR “anthocyanidin*” OR “procyanidin*” OR “flavonoid*”), b) (“food hypersensitivit*” OR “food allerg*” OR “allerg*”) AND (“MicroRNA*” OR “miR*”) AND (“mast cell*” OR “basophil*” OR “RBL-2H3*”), and c) (“MicroRNA*” OR “miR*”) AND (“mast cell*” OR “basophil*” OR “RBL-2H3*”) AND (“proanthocyanidin*” OR “anthocyanidin*” OR “ procyanidin*” OR “flavonoid*”). In PubMed, a) (“Food Hypersensitivity”[Mesh]) AND “MicroRNAs”[Mesh], b) (“Proanthocyanidins”[Mesh]) AND “MicroRNAs”[Mesh], c) (“Mast Cells”[Mesh]) AND “MicroRNAs”[Mesh], and d) (“Anthocyanins”[Mesh]) AND “MicroRNAs”[Mesh]. The reference lists of related articles were also searched to include relevant articles. Duplicate articles and research articles not in English were removed, and a total of 642 articles were screened. After title and abstract screening, 111 articles were selected, out of which 10 articles were found to be related to miRs and food allergies. Seven articles from the selected articles’ reference list were also found; thus, in total, 17 articles were included in this study (**Figure 3**).

### 2.2 Inclusion and exclusion criteria

Various study designs were included, including randomized clinical trials (RCTs), observational clinical trials, and *in vivo* studies published in English. Initially, databases with the aforementioned keywords were searched; articles based on the publication dates (since 2015), types of articles (original research articles only), and language (English) were adjusted in the searched websites; the duplicate articles were removed using MS Excel, leading to 642 articles. Of the 642, articles related to miRs expression studies on allergic diseases other than food or food compounds, food allergy studies that did not include miRs expression, and articles that included miRs expression and their role in immunological cell growth and development were excluded from the title and abstract screening, leading to 111 articles. These articles were analyzed in detail (full article) again to filter out based on criteria used for the 642 articles, leading to 10 articles, and these 10 articles were further searched for references to find the remaining seven research articles, leading to a total of 17 articles included in this study. The automation tools/tabs from each database searched were used to adjust articles based on the aforementioned inclusion and exclusion criteria. Two authors (T.S.R and J.P.R.) screened the initially selected 642 articles separately. Articles were selected based on the aforementioned inclusion and exclusion criteria (**Figure 3**), and any disagreements were resolved by consulting with other authors (R.R.B and L.L.W).

### 2.3 Data extraction and analysis

We extracted the following information from included clinical studies: a) study characteristics: reference, country, funding, study setting, and study design; b) population characteristics: allergy type, age, sex, history of allergy, and number of participants; and c) experimental characteristics: objectives, tissue/sample type, sample collection time, treatment vs. control, clinical sign measured, change in miRs expression, method of miRs expression analysis, molecule or gene target of the miRs, and confirmation method of potential targets of miRs. In the *in vivo* studies, we collected similar information to that of clinical studies except for the study setting, study design, and history of allergies. Two authors (T.S.R. and J.P.R.) extracted that information separately, discussed it with the third author (R.R.B.), and agreed upon the results. Since we had diverse studies in terms of participants (animal and human), intervention, control, outcome, and study design, we synthesized the results in a narrative review. We synthesized the results based on the food allergy types in clinical trials or in the *in vivo* (mouse) model.

### 2.4 Risk of bias assessment

All the RCTs were evaluated using the Cochrane Risk of Bias 2 (ROB 2) tool (42). Observational clinical trials were assessed according to the Newcastle-Ottawa scale. All animal studies were evaluated using SYRCLE’s risk of bias tool for animal studies guide (43) by two authors (T.S.R. and R.R.B).

## 3 Results

We followed the PRISMA checklist guidelines to conduct the study. After we removed duplicates and ineligible articles by automation (i.e., only research articles in English and keywords in the title and abstract), 642 articles remained. After a title and abstract review of 642 articles, 111 articles were left. Out of 111, only 10 articles remained after the removal of unrelated articles. We obtained seven articles from reference searching of 10 articles, resulting in 17 total articles for this review (**Figure 3**).

### 3.1 Risk of bias

All *in vivo* studies did not mention randomization and blinding processes, and most of them were free of selective reporting. Most of the clinical studies were observational studies with moderate quality based on the Newcastle-Ottawa scales. One clinical study was a completely randomized controlled design with a high risk of bias according to the ROB 2 method (see **Supplementary Material**).

### 3.2 Study characteristics

**Figure 4:**
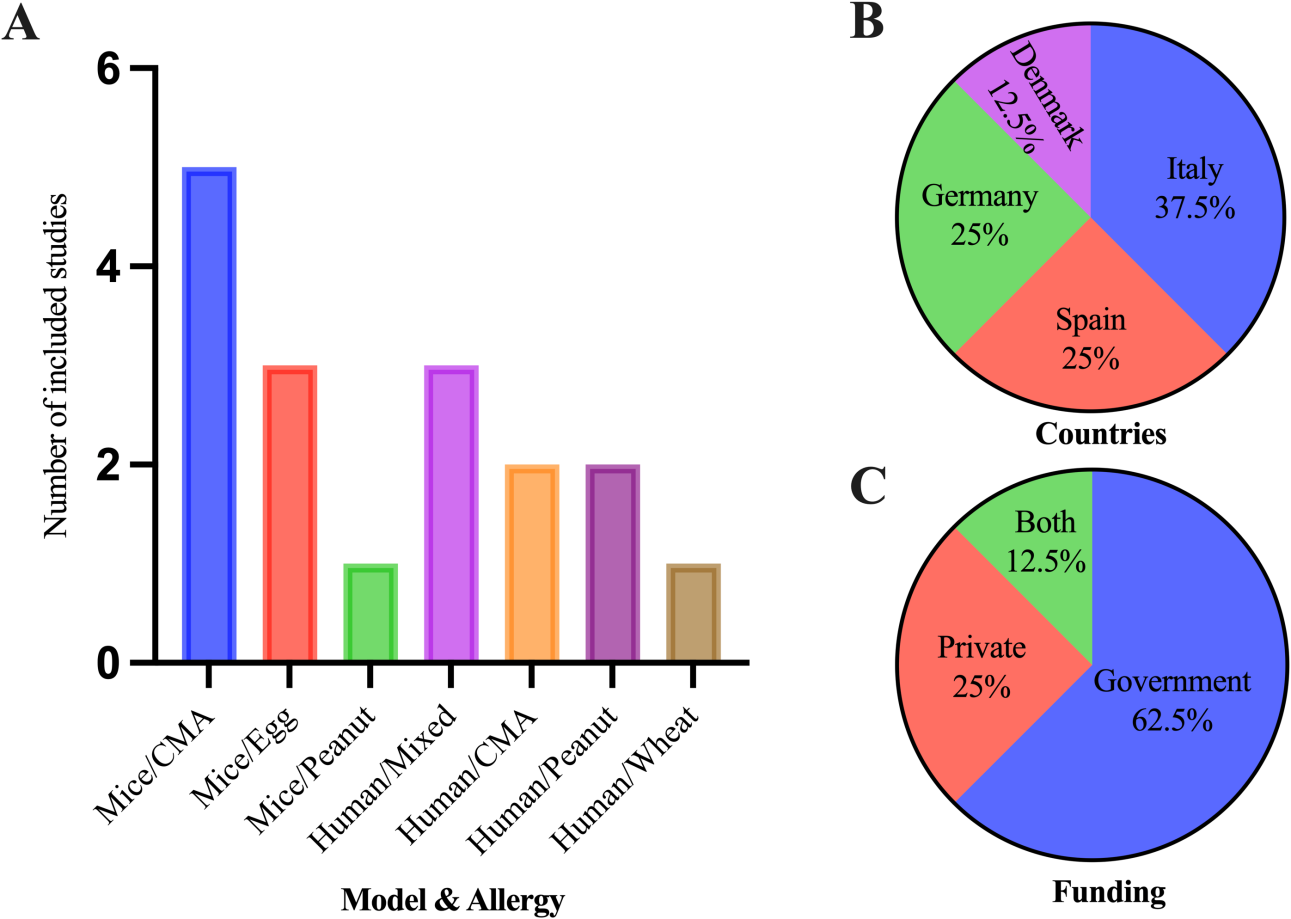
Characteristics of the included studies for the systematic review. **(A)** Number of studies included in both clinical and *in vivo* studies in different types of food allergies and miRs expression studies. **(B)** Countries where clinical trials of food allergies occurred. **(C)** Funding agencies of clinical trials for food allergies.

Out of eight clinical studies, three were on multiple food allergies, allergies to food and insect venom, or allergies to food and drugs. Both cow milk allergy (CMA) and peanut allergy had two studies each, and one study was related to non-celiac wheat sensitivity (NCWS) (**Figure 4A**). Five were cohort studies; one was an RCT, and two did not mention the study design. In a total of nine *in vivo* studies, five studies were on CMA (β-lactoglobulin), three studies were on egg [ovalbumin (OVA)] allergy, and one study was on peanut allergy. Clinical trials were primarily conducted in European countries: Italy, Spain, Germany, and Denmark (in the order of the highest to lowest number of studies) (**Figure 4B**). Of the included studies, 62.5% were government-funded, 25% were private company-funded, and 12.5% were funded by both private and government sectors (**Figure 4C**). All of the *in vivo* studies were conducted in China and funded by the government sector.

### 3.3 Population characteristics

Most clinical trials included either adults or children, and only one study included both population categories. However, both sexes (male and female) were included in all studies. The included participants experienced allergy either due to food challenges or accidental anaphylaxis, and some were included based on familial risk for allergy. Seven out of eight total studies tested miRs expression based on allergic versus non-allergic participants. In contrast, one study included the effect of diet intervention on miRs expression (**Table 1**). In the *in vivo* model, eight studies used BALB/c mice, and one used C57BL/6 mice. Each study included either 5–8-week-old female or male mice. Half of the studies tested miRs expression in allergic and non-allergic mice, and the rest included the intervention of diet (human milk oligosaccharides), probiotics (*Lactobacillus acidophilus*), or anti-inflammatory protein (galectin-1) (**Table 2**).

**Table 1:** Population characteristics of the participants in the included clinical trials.

Ref	Allergy type = participant number	Children/adults	Total participant number	Participants included in the analysis	Age (range)	Sex	History of allergy
Nuñez-Borque et al., 2021 (14)	Milk = 7, egg = 5, roosterfish = 2, peanut = 1, nut = 1, cashew = 1, cheese = 1, hazelnut = 1	Children	19	5 for both acute and basal phases with the most severe clinical symptoms used for NGS	Average = 11.3 (4–17 years)	Female = 11; male = 8	Anaphylaxis experienced due to food challenge
Nuñez-Borque et al., 2023 (15)	AF = 13, CF = 19, AD = 35 adults	Adults + children	67	35 AD = 5 NGS, 23 serum, 15 EVs; 13 AF = 10 serum, 8 EVs; 19 CF = 19 serum, 12 EVs	Average = 27.9 (4–76 years), AD = 44.6, AF = 33.3, CF = 10.5	Total (female = 60%; AD = 63%; AF = 54%; CF = 58%)	Anaphylaxis experienced accidentally
Francuzik et al., 2022 (44)	Venom = 22, food = 19, drug = 3, idiopathic = 3, healthy = 24	Adults	71	18	Female average = 47.08 (18–81 years); male average = 49.1 (23–78 years)	Female = 40	Anaphylaxis diagnosed in <3 hours
Paparo et al., 2019 (45)	CMA	Children	20 (10 on each type of diet)	20	EHCF + LGG = 6.5 months; SF = 7 months	Female = 50%	Familial risk of allergy (mother, father, siblings)
D’Argenio et al., 2017 (46)	CMA	Children	30 (10 CMA, 9 outgrown CMA, 11 healthy control)	NA	4–18 months	12 female (3 CMA, 6 healthy, 3 outgrown CMA)	Suspicion of CMA
Worm et al., 2022 (47)	PA	Adults	13 (6 PA and 7 non-allergic)	Allergic = 5, non-allergic = 4 for miR	>18 years (allergic male: 26–34, allergic female: 27–29; non-allergic female: 30–35, non-allergic male: 33)	Allergic female = 50%, non-allergic female = 85.7%	At least one severe allergic reaction in food challenge
Larsen et al., 2018 (48)	PA	Adults	24	Non-allergic = 10, allergic = 8	Non-allergic = 25 years (24–32), allergic = 20.5 years (19–29)	Female = 7 in non-allergic, female = 8 in allergic	PA patients based on clinical and positive oral food challenge
Clemente et al., 2019 (49)	NCWS	Adults	Pilot cohort (control = 17, NCWS = 13); validation cohort biopsies (control = 25, NCWS = 27, CD = 24); validation cohort PBL (control = 21, NCWS = 19)	NCWS = 13 + 27 (validation cohort), control = 17 + 25 (validation cohort)	Control = 51–55 years, NCWS = ~41 years, CD = ~39.5	Female > male	NA

AF, adult with food anaphylaxis; CF, children with food anaphylaxis; AD, anaphylaxis due to drug; CMA, cow milk allergy; PA, peanut allergy; NCWS, non-celiac wheat sensitivity; CD, celiac disease; PBL, peripheral blood leukocytes; NGS, next-generation sequencing; EVs, extracellular vesicles; EHCF, extensively hydrolyzed casein formula; LGG, *Lactobacillus rhamnosus* GG; NA, not available.

**Table 2:** Population characteristics of *in vivo* studies.

Ref	Mouse type	Age (weeks)	Weight (g)	Sex	Total number	Number included for analysis
Li et al., 2021 (50)	BALB/c	5–6	15–20	Female	48 (8 mice/group, 6 groups)	18 (3 mice/group in miRs)
Wang et al., 2018 (51)	BALB/c	5–6	23± 0.92	Female	30 (6 mice/group in 5 groups)	30 (6 mice/group in 5 groups)
Li et al., 2021 (52)	BALB/c	6–8	NA	Female	NA	NA
Zhang et al., 2020 (53)	BALB/c	5–6	NA	Female	30	30 (15 mice/group)
Wang et al., 2020 (54)	BALB/c	5–6	10	Female	20	20 (10 mice/group in two groups)
Yang et al., 2016 (55)	BALB/c	6–8	NA	Male	12	12 (6 mice/group in two groups)
Lin et al., 2017 (1)	BALB/c	6–8	NA	Male	24	24 (6 mice/group in four groups)
Liu et al., 2016 (56)	C57BL/6	6–8	NA	Male	NA	10 mice/group in miRs and IL-10; 6 mice/group for *in vivo* confirmation of miRs role
Xie et al., 2017 (57)	BALB/c	NA	NA	Male	24 (6 mice/group in 4 groups)	24

NA, not available.

### 3.4 Food allergy and miRs study in clinical trial

The clinical trials on food allergy included CMA, peanut allergy, NCWS, and mixed categories (food, drug, and venoms). The DNA was extracted from whole blood samples, serum, T cells, or peripheral blood-derived cells such as mononuclear cells, mast cells, or leukocytes (**Tables 3**–**6**). MiRs expression studies were conducted via next-generation sequencing (NGS) (five studies), microarray (two studies), or polymerase chain reaction (PCR) (one study). MiR-143-3p was commonly studied in peanut allergy and NCWS, and miR-191-5p and miR-3615 were commonly expressed in participants allergic to mixed food types (milk, fish, cheese, peanut, egg, cashew, and hazelnut) and milk (**Tables 3**–**6**).

**Table 3:** Findings and studies’ details of cow milk allergy (CMA) in clinical trials.

Ref	Study design	Allergy type	Objectives	Tissue/cells	Collection time	Treatment; control	Clinical sign measured	↑↓ of miRs	Validated miR among the miR list from NGS	Target and action test: *in vitro*/*in silico*	Target and action: result	NGS/PCR/microarray	MiR’s target confirmation (method; sample; cell lines; reagent; duration)
Paparo et al., 2019 (45)	Randomized control trial	CMA	Effect of two different diets on epigenetic mechanism of CMA	12 mL peripheral venous blood sample (>95% CD4^+^ T cells)	At enrollment, 6 months, and 12 months	EHCF+ LGG; SF	Prick test, gastrointestinal, vomiting, cutaneous, rash and urticaria, respiratory symptom, wheezing	EHCF + LGG ↑ miR-193a-5p, miR-146a, miR-128, miR-155 compared to SF	NA	PCR with correlation analysis	↑ MiR-128 ↓ IL-4, IL-5; ↑ miR-193a-5p ↓ IL-4; ↑miR-146a ↑ FoxP3; ↑miR-155 ↑ FoxP3 and ↓ IL-4	RT-PCR	NA
D’Argenio et al., 2017 (46)	Non-randomized clinical trial	CMA	Comparative study of miRome of children with CMA and healthy control	PBMC	During food challenge period (2 hours after final challenge)	CMA; healthy individuals	Gastrointestinal, cutaneous, respiratory	CMA ↓ miR-193a-5p, miR-197-3p, miR-423-5p, let-7b-5p, miR-486-5p, miR-92b-3p, let-7b-3p, miR-574-3p, miR-125a-5p, miR-93-3p, miR-3615, miR-423-3p, miR-320a, miR-191-5p, and ↑ miR-30a-5p and miR-224-5p	MiR-193a-5p was confirmed with RT-qPCR	*In silico*, qPCR	MiR-193a-5p ↓ IL-4 gene and protein	NGS, RT-PCR	HPB; human CD4^+^ T cells; transfection of miR-193a-5p inhibitor; 16 hours

miR, microRNA; CMA, cow milk allergy; PBMC, peripheral blood mononuclear cell; SF, soy formula; HPB, heparinized peripheral blood; ↓, decrease or inhibit; ↑, increase or promote; EHCF + LGG, extensively hydrolyzed casein formula with *Lactobacillus rhamnosus* GG; NGS, next-generation sequencing; RT-PCR, real-time polymerase chain reaction; NA, not available.

**Table 4:** Findings and studies’ details of peanut allergy in clinical trials.

Ref	Study design	Allergy type	Objectives	Tissue/cells	Collection time	Treatment; control	Clinical sign measured	↑↓ of miRs	Validated miR among the miR list from NGS	Target and action test: *in vitro*/*in silico*	Target and action result	NGS/PCR/microarray	MiR’s target confirmation (method; sample; cell lines; reagent; duration)
Worm et al., 2022 (47)	Non-randomized clinical trial	PA	Mechanism of PA	Blood + stool	Before oral food challenge for all tests (including microbiome and epigenetics), for ELISA and miR sequencing before and 1 hour after oral food challenge	PA patients; non-allergic patients	Atopic dermatitis, asthma, rhinitis, tryptase	PA ↑ 15 miRs: miR-574-5p, miR-4669, miR-9901, miR-3621, miR-4299, miR-10400-5p, miR-3960, miR-4787-5p, miR-3665, miR-3195, miR-718, miR-10394-3p, miR-4488, miR-1908-3p, miR-143-3p	MiR-143-3p and miR-718 were validated with qCPR	NA	NA	NGS, RT-PCR	NA
Larsen et al., 2018 (48)	Non-randomized clinical trial	PA	Investigate molecular and stimulus-response profile of human PBdMC of PA and non-allergic individual	PBdMC	NA	Peanut-allergic patients; non-allergic subjects	Asthma, other food allergies in 63% (5/8) of allergic patients	No difference in miRs profile between treatment and control	NA	NA	NA	miR arrays	NA

miR, microRNA; PA, peanut allergy; PBdMC, peripheral blood-derived mast cell; ↓, decrease or inhibit; ↑, increase or promote; NGS, next-generation sequencing; RT-PCR, real-time polymerase chain reaction; NA, not available.

**Table 5:** Findings and studies’ details of non-celiac wheat sensitivity (NCWS) in clinical trials.

Ref	Study design	Allergy type	Objectives	Tissue/cells	Collection time	Treatment; control	Clinical sign measured	↑↓ of miRs	Validated miR among the miR list from NGS	Target and action test: *in vitro*/*in silico*	Target and action result	NGS/PCR/microarray	MiR’s target confirmation (method; sample; cell lines; reagent; duration)
Clemente et al., 2019 (49)	Non-randomized clinical trial	NCWS	Expression level of miRs in duodenal biopsies and blood with NCWS and celiac disease and gluten-independent gastrointestinal problems	DB and PBL	NA	NCSW patients; patients with celiac disease and gluten gastrointestinal problems	NA	In both NCSW patient’s DB and PBL, ↑ miR-19b-3p, miR-19a-3p, miR-186-5p, miR-17-5p, miR-145-5p, miR-30e-5p, and ↑ miR-143-3p in DB	NA	NA	NA	mRNA assay	NA

NCWS, non-celiac wheat sensitivity; DB, duodenal biopsy; PBL, peripheral blood leukocyte; ↓, decrease or inhibit; ↑, increase or promote.

**Table 6:** Findings and studies’ details of food, drug, and insect venom in clinical trials.

Ref	Study design	Allergy type	Objectives	Tissue/cells	Collection time	Treatment; control	Clinical sign measured	↑↓ of miRs	Validated miR among the miR list from NGS	Target and action test: *in vitro*/*in silico*	Target and action: result	NGS/PCR/microarray		MiR’s target confirmation (method; sample; cell lines; reagent; duration)
Nuñez-Borque et al., 2021 (14)	Non-randomized clinical trial	Milk, egg, others (roosterfish, peanut, nut, cashew, cheese, and hazelnut)	MiRs profile in anaphylaxis, miRs as biomarker, and miRs participation in molecular mechanism in anaphylaxis	Blood	Acute phase = first 30 minutes of start of reaction; basal phase = 14 days after the reaction	AFA children; BFA children	Symptoms on skin, digestive system, and respiratory system (rhinitis). Signs such as heart rate, oxygen saturation, and tryptase activity	41 miRs differentially expressed between acute and basal phase. Acute phase ↑ 21 miRs: miR-21-3p, miR-199b-5p, miR-509-3p, miR-424-3p, miR-1299, miR-95-3p, miR-99b-3p, miR-487b-3p, miR-625-5p, miR-452-5p, miR-185-3p, miR-127-3p, miR-331-3p, miR-3688-3p, miR-191-5p, miR-181a-2-3p, miR-326, miR-29c-3p, miR-29c-5p, miR-181a-5p Acute phase ↓ 20 miRs: miR-6741-5p, miR-92a-3p, miR-3615, miR-1260a, miR-144-5p, miR-4433b-5p, miR-3173-5p, miR-181c-5p, miR-551a, miR-550a-3p, let-7a-3p, miR-636, miR-2276-3p, miR-214-3p, miR-483-3p, miR-642a-3p, miR-6805-5p, miR-34c-5p, miR-202-3p, miR-433-3p	In acute FA, ↑ miR-21-3p and miR-487b-3p compared to basal allergy of all 19 participants	*In silico*	MiR-21-3p associated with TRL signaling, and B-cell activated factor signaling, and miR-487b-3p associated with role of JAK2 in hormone-like cytokine signaling, histamine degradation, and IL-9 signaling; both miRs were associated with cancer. miR-487-3p and miR-21-3p were associated with inflammatory disease and dermatological diseases, respectively	NGS, RT-PCR		qPCR; serum; transfection of HMVEC-D; cocktail of mediators (histamine, PAF, and thrombin) OR serum from acute phase anaphylaxis; 2 hours
Nuñez-Borque et al., 2023 (15)	Non-randomized clinical trial	Anaphylaxis to food and drug	MiRs’ involvement in anaphylactic reaction and their value as biomarkers	Blood	Basal phase = 14 days after reaction	Acute anaphylaxis; basal anaphylaxis	Cutaneous, mucosal, gastrointestinal, respiratory, neurological, and cardiovascular	21 miRs were differentially expressed. Acute phase ↑ 7 miRs: miR-211-5p, miR-6513-3p, miR-4446-3p, miR-6852-5p, miR-139-5p, miR-133a-3p, miR-326 Acute phase ↓ 14 miRs: miR-320c, miR-1226-3p, miR-205-5p, miR-885-5p, miR-3173-5p, miR-206, miR-203a, miR-122-5p, miR-375-3p, miR-1247-5p, miR-193b-5p, miR-3150b-3p, miR-4685-3p, miR-885-3p	Acute anaphylactic phase patients had ↓ miR-375-3p	*In silico*	↓ of miR-375-3p associated with ↑ MCP-1 and GM-CSF, and ↓ Rac1-Cd42	NGS		qPCR: serum and extracellular vesicles; HMVEC-D transfection (*in vitro* endothelial assay); miR-375-3p over-expression; endothelial resistance at 30 and 45 mins of transfection
Francuzik et al., 2022 (44)	Non-randomized clinical trial	Insect venom, food, drug	Serum biomarker profiles (proteins, lipids, and miRs) for diagnosis of anaphylaxis	Blood	<3 hours of anaphylaxis and 1 month later of the reaction	Anaphylactic individuals; healthy individuals	Skin symptoms, respiratory, cardiovascular, and gastrointestinal symptoms	Anaphylaxis ↑ miR-451a	MiR-451a	NA	NA	NGS, RT-PCR	NA	

miR, microRNA; AFA, acute food allergy; BFA, basal food allergy; MCP-1, monocyte chemoattractant protein-1; JAK2, Janus kinase 2; GM-CSF, granulocyte macrophage colony-stimulating factor; HMVEC-D, human dermal microvascular endothelial cells; PAF, platelet-activating factor; ↓, decrease or inhibit; ↑, increase or promote; NGS, next-generation sequencing; RT-PCR, real-time polymerase chain reaction; NA, not available.

#### 3.4.1 Cow milk allergy

Two studies were conducted on children aged 6-18 months. The first study was on the effect of diet (excessively hydrolyzed casein formula + *Lactobacillus rhamnosus* GG vs. soy formula) on CMA (45). In contrast, the second study compared the miRs expression in CMA children versus healthy children (46). Both studies analyzed peripheral blood samples generating CD4^+^ T cells and peripheral blood mononuclear cells (PBMCs). Both studies determined that CMA reduced miR-193a-5p, which was negatively correlated with IL-4, while extensively hydrolyzed casein formula (EHCF) + *L. rhamnosus* GG (LGG) increased this miR. The effect of miR-193-5p on targeting molecules was further tested in *in vitro* by RNA interference (RNAi) in human CD^+^ T cells (46). Furthermore, EHCF + LGG increased other miRs such as miR-128, which was negatively correlated to IL-4 and IL-5; miR-146a, which was positively correlated to FoxP3; and miR-155, which was positively correlated to FoxP3 and negatively correlated to IL-4 (45) (**Table 3**).

#### 3.4.2 Peanut allergy

Two studies were on miRs expression in the peanut-allergic group versus the non-allergic group of 26–33-year-old adults. In one study, peanut allergy increased the expression of 15 miRs: miR-574-5p, miR-4669, miR-9901, miR-3621, miR-4299, miR-10400-5p, miR-3960, miR-4787-5p, miR-3665, miR-3195, miR-718, miR-10394-3p, miR-4488, miR-1908-3p, and miR-143-3p. However, only two were validated with qPCR: miR-143-3p and miR-718 (47). The second study found no differentially expressed miRs between allergic and non-allergic participants (48) (**Table 4**).

#### 3.4.3 Non-celiac wheat sensitivity

The miRs expression between NCWS and celiac disease and gluten-independent gastrointestinal problems in adults (40–55 years) was studied via miR array (49). NCWS increased miR-19b-3p, miR-19a-3p, miR-186-5p, miR-17-5p, miR-145-5p, and miR-30e-5p in blood and duodenal biopsies. The target molecules of miRs need to be determined (**Table 5**).

#### 3.4.4 Food allergies, along with insect venom or drugs

The first study included only food allergy in children (4–17 years) (14), the second study included food and drug allergy in adults (33–45 years) (15), and the third one included food, venom, and drug allergy in adults (18–81 years) (44). Blood samples were collected within 30 minutes to 3 hours of anaphylaxis for the acute phase and 14 days to 1 month later for the basal phase. Anaphylaxis due to food increased the expression of miR-21-3p and miR-487b-3p (14), anaphylaxis due to food and drug reduced the miR-375-3p expression (15), and anaphylaxis due to food, venom, and drugs increased the expression of miR-451a (44). These miRs were validated via qPCR after their differential expression was analyzed via NGS. MiR-21-3p was associated with Toll-like receptor (TLR) signaling and B-cell activation factor signaling, while miR-487b-3p was associated with JAK2’s role in cytokine signaling, histamine degranulation, and IL-9 signaling. Molecular targets of the validated miRs were conducted by *in silico* analysis and also via *in vitro* experiments either treating with a cocktail of serum of acute anaphylactic patients (14) or over-expressing miR-375-3p in human dermal microvascular endothelial cells (HMVEC-D) (15) (**Table 6**).

### 3.5 Food allergy and miRs study in *in vivo* model

The *in vivo* studies included CMA, egg allergy, and peanut allergy. The DNA was extracted from the colon, intestine, spleen, or specific types of cells such as B cells and macrophages. All studies used PCR for miRs expression analysis except one (which used NGS). All but one study used BALB/c mice aged 5–8 weeks, five studies used only female mice, and four used only male mice (**Table 2**). MiR-155 was found commonly in both milk (β-lactoglobulin) and egg (OVA) allergies (**Tables 7**, **8**).

**Table 7:** Findings and studies’ details of cow milk allergy (CMA) in *in vivo* studies.

Ref	Objectives	Allergy type	Tissue/cells	Sample collection time	Treatment; control	Administration route	Treatment dose	Treatment duration	↑↓ of miRs	MiR’s target and action	NGS/PCR/microarray	MiR’s target test: *in vitro*/*in silico*	Confirmation of miR’s target result (method; cell lines; reagent and dose; duration)
Li et al., 2021 (50)	Molecular mechanism of HMO and its component 2′-FL on intestinal immunity against CMA	*β*-LG	Colonic (miRs) and auricle tissue	1 hour after last oral challenge of *β*-LG	2′-FL + *β*-LG (low, medium, and high), HMO + *β*-LG; *β*-LG (+ve), sterile saline (−ve)	Intraperitoneal = *β*-LG; oral gavage = HMO, 2′-FL	*β*-LG at the rate of 0.2 mL of 1 mg/mL/mouse. HMO at the rate of 400 mg/kg bwt, 2′-FL at the rate of 200–600 mg/kg bwt, or sterile saline (control group)	*β*-LG on 7, 14, and 21 days. HMO and 2′-FL 3 times/week from day 1 to day 28	2′-FL ↑ miR-146a	MiR-146a ↓ TLR4/NF-κB pathway	RT-PCR	*In vitro*	RNAi via miR-146a inhibitor ‘s transfection; RAW264.7 cells; Lipofectamine 2000 reagent at the rate of 100 nM; 48 hours
Wang et al., 2018 (51)	Correlation between *Lactobacillus acidophilus* to modulate miRs and prevent Th17-dominated *β*-lactoglobulin allergy	*β*-LG	Blood, spleen and colon tissues, and feces	28 days after treatment started	*L. acidophilus*; *β*-LG	Intraperitoneal = *β*-LG; intragastric = *L. acidophilus*; oral = *L. acidophilus* suspension; oral = *β*-LG challenge	*β*-LG at the rate of 0.2 mL of 1 mg/mL *β*-LG dissolved in Freund’s adjuvant per mouse of *β*-LG and *L. acidophilus* treatment. *L. acidophilus* suspension at the rate of 0.2 mL of 5 mL/kg wt. *β*-LG challenge at the rate of 20 mg/mouse	*β*-LG on 7, 14, and 21 days. *L. acidophilus* suspension 3 times/week from day 1 to day 28. *β*-LG oral challenge on day 28	*β*-LG ↑ and *L. acidophilus* ↓ miR-146a, miR-155, miR-21, miR-9	↓ miR-146a, miR-155, miR-21, and miR-9 associated to ↓ IL-17 and RORγt mRNA	RT-PCR	NA	NA
Li et al., 2021 (52)	Effects of probiotics on regulation of TLR4/NF-κB pathway and miRs expression in *β*-LG macrophages	*β*-LG	Peritoneal lavage fluid (for macrophage harvest)	Macrophages collected (from PLF) of *β*-LG treated group	*L. acidophilus* strains; *β*-LG (+ve), saline (−ve).	Sensitization: intraperitoneal. Challenge: oral gavage. Macrophages of *β*-LG treated group were exposed to *β*-LG	Sensitization: *β*-LG at the rate of 0.2 mL of *β*-LG dissolved in Freund’s adjuvant at the rate of 1 mg/mL. Challenge: *β*-LG at the rate of 20 mg/mouse twice. Macrophage exposure: *β*-LG at the rate of 1 mg/mL with or without *L. acidophilus* strains at the rate of 10^7^ CFU/mL or 10 μM TLR4 inhibitor C34	Sensitization: on days 7, 14, and 21. Challenge: on day 28. For macrophage: *β*-LG treatment for 24 hours	*β*-LG ↑ TLR4/NF-κB. *L. acidophilus* La KLDS 1.0738 ↑ miR-146a	MiR-146a ↓ TLR4 pathway	RT-PCR	*In vitro*	RNAi via transfection of 50 nM miR-146a inhibitor; Macrophage; 36 hours
Zhang et al., 2020 (53)	Difference in colonic miRs profile of *β*-LG allergic and normal mice	*β*-LG	Blood, colon tissue (miRs)	On day 28, 2 hours after challenge	*β*-LG; saline (−ve)	Sensitization: intraperitoneal. Challenge: oral gavage	Sensitization: *β*-LG at the rate of 0.2 mL dissolved at the rate of 1 mg/mL in Freund’s adjuvant. Challenge: *β*-LG at the rate of 20 mg/mouse twice	Sensitization = 21 days. Challenge = On day 28	*β*-LG ↑ miR-19a-5p, miR-365-2-5p, miR-504-5p, miR-1193-3p, miR-224-5p, miR-106a, and *β*-LG ↓ miR-30a-5p, miR-150-5p, oan-miR-194-5p, bta-miR-150, miR-7977, miR-4454, miR-433-3p, miR-669, miR-1934-5p	MiR-19a-5p↓ PTEN, SOCS1, and Tgfbr2, miR-365-2-5p ↓ FoxP3, miR-504-5p ↓ FoxP3, and miR-224-5p ↓ Tgfbr2	NGS, RT-PCR. miR validated with RT-qPCR: miR-224-5p, miR-365-2-5p, miR-504-5p, miR-19a-5p, miR-150-5p, miR-30a-5p, miR-106a-5p	*In silico* + *in vitro*	RNAi via transfection with 60 nM miR-19a mimics and inhibitors; RAW264.7; 36 hours
Wang et al., 2020 (54)	Expression of miRs and Th17 cells in *β*-LG allergy	*β*-LG	Blood, lungs, colon spleen (miRs from colon and spleen)	On day 16, at 0.5, 1, 1.5, and 3 hours after challenge	*β*-LG; saline (−ve)	Sensitization: intraperitoneal. Challenge: oral gavage	Sensitization: *β*-LG at the rate of 1 mL Freund’s adjuvant + 1 mL of 1 mg/mL *β*-LG. Challenge: *β*-LG at the rate of 5 mg/mouse	Sensitization: on days 1, 7, and 14. Challenge: on day 16	*β*-LG ↑miR-146a and miR-155 in colon, spleen, and splenic lymphocyte	As miR-146a and miR-155 ↑, IL-17 and its transcription factor RORγt (in spleen) and also ↑	RT-PCR	*In vitro*	RNAi with shRNA or control shRNA of miR-19a; B cells

HMO, human milk oligosaccharides; 2′-FL, 2′-fucosyllactose; CMA, cow milk allergy; *β*-LG, *β*-lactoglobulin; +ve, positive control; −ve, negative control; PLF, peritoneal lavage fluid; TLR4, Toll-like receptor 4; NF-κB, nuclear factor kappa-B; RNAi, RNA interference; RORγt, retinoic acid receptor-related orphan nuclear receptor gamma; PTEN, phosphatase and tensin homolog; SOCS1, suppressor of cytokine signaling 1; Tgfbr2, transforming growth factor beta receptor type 2; FoxP3, forkhead box protein P3; shRNA, short hairpin RNA; ↓, decrease or inhibit; ↑, increase or promote; NGS, next-generation sequencing; RT-PCR, real-time polymerase chain reaction; bwt, body weight; NA, not available.

**Table 8:** Findings and studies’ details of egg allergy (OVA) in *in vivo* studies.

Ref	Objectives	Allergy type	Tissue/cells	Collection time	Treatment; control	Administration route	Treatment dose	Treatment duration	↑↓ of miRs	miR’s Target and Action	NGS/PCR/microarray	miR’s Target test: *in vitro*/*in silico*	Confirmation of miR’s target result (method; cell lines; reagent and dose; duration)
Yang et al., 2016 (55)	MiR-17–92 cluster’s role in TSP1 regulation in intestinal CD35+ B cells	OVA	Blood, intestine (CD35+ B cells, CD4+ T cells)	One day after week 5 (food challenge)	OVA; normal food	Sensitization and challenge: gavage-fed	Sensitization and challenge: OVA at the rate of 1 mg/mouse + CT at the rate of 20 μg/mouse in 0.3 mL saline	Sensitization: once/week for consecutive 5 weeks. Challenge: week 5	OVA ↑ miR-19a in B cells	As miR-19a ↑, TSP1 protein in B cells↑	RT-PCR	NA	NA
Lin et al., 2017 (1)	Effect of miR-155 on B10 function to facilitate the intestinal food allergy	OVA	Blood, intestine (B10 cell)	60 minutes after challenge, i.e., immediately after sacrifice	OVA; normal food	Sensitization and challenge: gavage-fed	Sensitization: OVA at the rate of 1 mg/mouse + CT at the rate of 20 μg/mouse in 0.3 mL saline. Challenge: OVA at the rate of 5 mg/mouse	Sensitization: once/week for 4 consecutive weeks. Challenge: week 5	OVA ↑ miR-155 in CD19^+^ B cell	MiR-155 ↑ IL-13 and ↓ IL-10 (in CD19^+^ B cells)	RT-PCR	*In vitro*	RNAi; B cells
Liu et al., 2016 (56)	Role of miR-17–92 cluster in food allergen-related inflammation in intestine	OVA	B cells (for miRs, IL-10 mRNA) and CD4^+^ T cells from small intestine; blood (for IL-4 and IgE test); jejunum tissue (eosinophil infiltration)	1 week after the last OVA treatment (up to 4 weeks after establishment)	OVA; normal food	Oral gavage	OVA at the rate of 1 mg/mouse. CT at the rate of 20 μg/mouse in 0.3 mL saline	Weekly for 4 weeks	OVA↑ miR-19a	MiR-19a ↓ IL-10	RT-PCR	*In vitro*	MiR-19a RNAi and *in vivo* experiment on miR-19a-deficient mice; B cells; normal mice vs. miR-19a-deficient mice; intraperitoneal injection of LPS at the rate of 10 mg/kg bwt and/or IL-4 at the rate of 4 mg/kg bwt; 5 days

OVA, ovalbumin; TSP1, thrombospondin-1; CT, cholera toxin; B10, regulatory B cell; RNAi, RNA interference; LPS, lipopolysaccharide; ↓, decrease or inhibit; ↑, increase or promote; NGS, next-generation sequencing; RT-PCR, real-time polymerase chain reaction; bwt, body weight; NA, not available.

#### 3.5.1 Cow milk (β-lactoglobulin) allergy

In BALB/c mice (female), the effect of human milk oligosaccharides (HMOs) and fucosyllactose (2′-FL) (50), *L. acidophilus* strains’ effect on CMA (51, 52), or difference on miRs between CMA and non-allergic mice were studied (53, 54). After sensitization for 21 days and challenging on the 28th day (except in one study) (54), the expression of selected miRs was assessed. MiR-146a (50–52, 54) and miR-155 (51, 54) were commonly expressed in most studies. MiR-146a had a negative association with TLR4/NF-κB signaling (52) and a positive association with IL-17 and RORγt (51, 54). Moreover, miR-9 was also positively associated with IL-17 and RORγt (54), and miR-19a was negatively associated with PTEN, Tgfbr4, and SOCS1. Both miR-365–2 and miR-504 were negatively associated with FoxP3, and miR-224 reduced Tgfbr2 (53). The targets of the miRs were validated via RNAi in B cells (54), macrophage (52), or RAW 264.7 (50, 53) (**Table 7**).

#### 3.5.2 Egg (OVA) allergy

The miRs profile of allergic and non-allergic male BALB/c (1, 55) or C57BL/6 (56) mice were studied by sensitizing them with the oral treatment of OVA (1 mg/mouse) + cholera toxin (20 μg/mouse) for 4-5 weeks and challenged with OVA (1 or 5 mg/mouse). Allergies increased the expression of miR-19a and miR-155 in B cells. Both miRs reduced IL-10 (1, 56), miR-19a increased thrombospondin-1 (TSP1) (55), and miR-155 increased IL-13 (1). The target molecules of the miRs were validated via RNAi in B cells and in *in vivo* with miR-19a-deficient mice (**Table 8**).

#### 3.5.3 Peanut allergy

The role of galectin-1 on intestinal allergy was studied in BALB/c male mice by sensitizing them until 14 days, three times/day, and challenged (5 mg/mouse) on the 15th day. Galectin-1 reduced miR-98, and miR-98 increased IL-4 and reduced IL-10. The target molecules of miRs was validated via RNAi of CD14^+^ cells isolated from lamina propria mononuclear cells (LPMCs) (57) (**Table 9**).

**Table 9:** Findings and studies’ details of peanut allergy in *in vivo* studies.

Ref	Objectives	Allergy type	Tissue/cells	Collection time	Treatment; control	Administration route	Treatment dose	Treatment duration	↑↓ of miRs	MiR’s target and action	NGS/PCR/microarray	MiR’s target test: *in vitro*/*in silico*	Confirmation of miR’s target result (method; cell lines; reagent and dose; duration)
Xie et al., 2017 (57)	Gal-1 inhibits oral-intestinal allergy syndrome	PA	Blood, buccal mucosa, small Intestine	1 day after last day of treatment (14 days)	Gal-1 + PE; PE	Sensitization: buccal mucosa; PE challenge: gavage	Sensitization with PE: CT (10:1, w/w) and PE: Gal-1: CT (10:10:1, w/w/w). Challenge: PE at the rate of 5 mg/mouse	Sensitization: up to 14 days at the rate of 3 times/day. Challenge: on day 15	Gal-1 ↓ miR-98	MiR-98 ↑ IL-4 and ↓ IL-10	RT-PCR	*In vitro*	RNAi via transduction with CD45 shRNA-carrying lentivirus; CD14^+^ cells

PA, peanut allergy; Gal-1, galectin-1; PE, peanut protein extract; CT, cholera toxin; RNAi, RNA interference; shRNA, short hairpin RNA; RT-PCR, real-time polymerase chain reaction; ↓, decrease or inhibit; ↑, increase or promote.

## 4 Discussion and future perspectives

**Figure 5:**
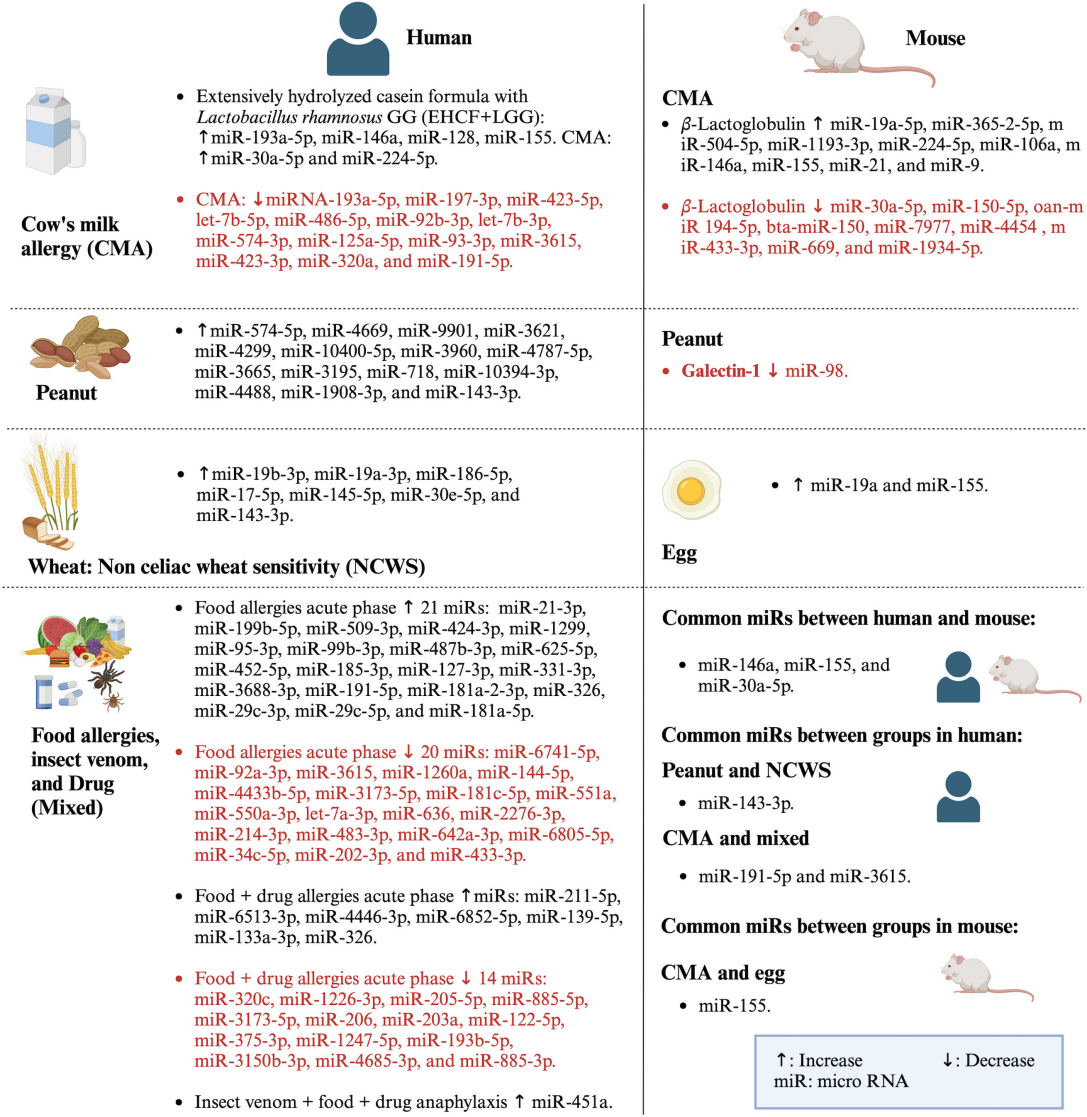
Summary of miRs studied in both clinical trials and *in vivo* studies along with common miRs expressed between and within the studies. Created with BioRender.com.

The exclusion of miR studies related to allergies other than food allergies led to the determination of very few common miRs signatures among food allergies. However, those commonly studied miRs were also studied in other allergic as well as non-allergic diseases. We selected some of the miRs from this study for discussion based on the following criteria: a) miRs commonly expressed within or between clinical trials and *in vivo* studies, b) miRs whose target molecules were tested, c) miRs those were selectively used in the included studies based on results of previous studies, and d) miRs obtained from NGS and validated with PCR. In this study, the miRs commonly studied in both clinical trials and *in vivo* studies were miR-146a, miR-155, and miR-30a-5p (**Figure 5**). These miRs were also studied in other allergic diseases. For example, miR-146a, which was increased in CMA (50–52, 54), was reported to reduce allergic rhinitis (58–60), atopic dermatitis (61), and allergic asthma (62). MiR-155, which was increased in CMA (51, 54) and egg allergy (1), had a positive association with allergic rhinitis (63) and eosinophilic inflammation (64) and a negative association with allergic asthma (65). Moreover, miR-30a-5p, which was increased in CMA in a clinical trial (46) and decreased in CMA in an *in vivo* study (53), had a negative association with asthma (66) and a positive association with allergic rhinitis (67).

In addition to the aforementioned miRs, other miRs were also commonly studied. In clinical trials, miR-143-3p expression was increased in peanut (47) and wheat allergy (49). MiR-191-5p increased in both CMA and food allergy, and miR-3615 increased in CMA and decreased in food allergy. These miRs also related to other allergic and non-allergic diseases. For instance, miR-143-3p (68) and miR-191-5p (69) had negative and positive, respectively, regulation on allergic asthma. MiR-3615 was studied less in allergic diseases but was found to be positively correlated with other diseases such as hepatocellular carcinoma (70). Similarly, other miRs tested and validated with PCR in CMA, peanut allergy, and food allergy were found to be expressed in other diseases too. For example, miR-128 reduced in CMA (45) was positively associated with asthma (71), and miR-193a-5p, which had reduced (46) or enhanced (45) expression in CMA, also enhanced respiratory distress (72). MiR-21-3p increased in food allergy (14) also positively correlated with asthma (73). The miR-375-3p, which was reduced in food allergy (15), also negatively regulated atopic dermatitis (74). MiR-451a, which was increased due to allergy to food, venom, and drugs (44), reduced allergic asthma (75, 76). The miR-487b-3p was increased in food allergy (14) and reduced in allergic asthma (77, 78). The increased miR-718 in peanut allergy (47) was reported to inhibit autoimmune disease such as psoriasis (79).

In *in vivo* studies, miRs were expressed in various allergies. For instance, in CMA, miR-21, miR-9, miR-365-2-5p, miR-504-5p, miR-224-5p, and miR-106a were increased and miR-150-5p was decreased (51, 53); and miR-19a and miR-98 were increased in egg and peanut allergy, respectively (55, 57). These miRs were also studied in other allergic diseases. For example, miR-21 negatively regulated allergic rhinitis (80, 81) and positively regulated bronchial asthma (82) and eosinophilic esophagitis (83). MiR-9 was increased in neutrophilic asthma (84) and reduced in atopic dermatitis (85). MiR-365-2-5p promoted osteogenesis (86), and miR-504-5p suppressed glioblastomas (87). MiR-224-5p (88–90) and miR-106a (58–60) alleviated allergic rhinitis and atopic dermatitis (61). Moreover, miR-150-5p was found to increase allergic rhinitis (91) and rhinosinusitis (92). MiR-19a (93, 94) and miR-98 (95, 96) increased asthma and rhinosinusitis (97). These all reports and discussions indicate that the same miRs could have different effects in different physiological conditions/diseases, which may be because the same miRs can have different molecular targets for different diseases or physiological conditions. For instance, in the clinical trial of CMA, miR-146a had a positive association with FoxP3 (45), but in *in vivo* studies, it had a negative association with TLR4/NF-κB (50, 52) and a positive association with RORγt and IL-17 (51, 54). Moreover, miR-155, which positively regulated FoxP3 and IL-4 in clinical trials of CMA (45), also positively regulated RORγt, IL-17, and IL-13 and negatively regulated IL-10 in CMA and egg allergy (1, 51, 54). Thus, these evidences also imply that studying each miR for the specific context of food allergy is crucial before generalizing them as a common miR for food allergy.

There are both similarities and dissimilarities in the results of miRs expression studies included in this review and the findings of studies on the potential role of the miRs on immune cells or molecules involved in allergies. For example, miR-155, which was increased due to CMA in *in vivo* studies (51, 54), was reported to enhance the maturation and function of DCs, promote B-cell response, promote Tfh cells, and promote the conversion of B cells into plasma cells (**Figure 1**). Similarly, miR-9 and miR-19a were increased in CMA (51) and OVA allergies (55, 56), respectively, in *in vivo* studies, which were determined to increase the maturation and function of DCs and promote Th2 cells, respectively (**Figure 1**). Moreover, miR-21 was increased in CMA in *in vivo* studies (51) and was also reported to promote Th2 production and DC differentiation (**Figure 1**). Furthermore, let-7b-3p and let-7b-5p were reduced in CMA in clinical trials (46) and were mentioned to inhibit IL-13 production (**Figure 1**). However, miR-146a, which was increased in both *in vivo* studies (51, 54) and clinical trials (45), was found to inhibit proinflammatory cytokine and cells (IL-5 and Tfh13) (**Figure 1**). Moreover, miR-155 and miR-21 were also reported to reduce the Th2 production and activation of mast cells and macrophages, respectively (**Figure 1**). These dissimilarities in results may be due to differences in the studies parameters such as results from *in vitro* versus *in vivo* versus clinical trials. There is a need for further studies on the modulatory effect of differentially expressed miRs on immune cells and molecules involved in food allergies.

There are various limitations of this study. For example, most of the clinical studies included are observational studies; thus, the method used for their quality evaluation is a subjective approach based on the information provided in the articles and the supplementary materials. Moreover, some of the included clinical trials combined multiple food allergies or food allergies with drug or insect venom allergies, limiting the finding of these studies useful for specific food allergy-related signature miRs. Most animal studies were related to β-lactoglobulin, followed by OVA and peanut allergy. Furthermore, all of the mouse model studies were based on one country, i.e., China. Thus, more studies in different geographical areas targeting a particular type of food allergy are required to increase reliable miRs signatures and their molecular targets in food allergy. Moreover, in *in vivo* studies, one study used NGS for miRs profiling, and the rest of the studies used the previously studied miRs and quantified by PCR method. This may also led to a few common miRs shared between clinical trials and *in vivo* studies (**Figure 5**). Furthermore, more target molecules of differentially expressed miRs were studied in *in vivo* studies compared to those of clinical trials due to more available references on target molecules of the selected miRs. However, in clinical trials, half of the included studies did not focus on target molecules of differentially expressed miRs, again leaving more work to be conducted for molecular targets study of the miRs for them to be a reliable marker for a specific food allergy.

Because few studies were available on a particular food allergy, such as in peanut allergy and its related miRs expression, it is hard to generalize different signature miRs for a specific type of food allergy. Moreover, very few miRs are common among various kinds of food allergies within clinical trials or within *in vivo* studies. It indicates that more miR target-specific studies and studies specific to a particular food allergy are still required in clinical studies. In contrast, in *in vivo* studies, novel miRs may need to be explored via NGS. Additionally, the differentially expressed miRs via NGS are required to be validated via PCR, and those validated miRs need to be further studied for their target molecules with additional confirmation either in *in vitro* method via RNAi (e.g., transfection) or in *in vivo* method via target gene knockout/knockdown model.

In conclusion, very few common miRs were studied/expressed between or within the clinical trials and *in vivo* studies or within an individual study of food allergy. Moreover, the same miR can have different molecular targets and effects on specific diseases; thus, it is essential to study miRs and their targets in particular contexts of food allergy, such as peanut allergy, egg allergy, or β-lactoglobulin allergy, to determine their therapeutic target for each type of food allergy. Furthermore, future studies of food allergy should focus on the identification of novel miRs via NGS, their validation via PCR, and miR’s target molecules determination. This may increase the possibility for signature miRs identification for an individual food allergy type as well as for food allergy in general.

## Data Availability

The original contributions presented in the study are included in the article/**Supplementary Material**. Further inquiries can be directed to the corresponding author.
