# Supplementary material for: MicroRNA expression and their molecular targets in food allergies: a systematic review
Source: Front Immunol. 2025 May 12;16:1524392. doi: 10.3389/fimmu.2025.1524392 (PMC12104090; doi:10.3389/fimmu.2025.1524392)
Supplement: Supplementary file 1 [file Table1.docx]

Supplementary Material

Supplementary Table 1. Risk of bias evaluation of *in vivo* studies with SYRCLE method (1) .

| **Ref** | **Was the allocation sequence adequately generated and applied**  **? (1)** | **Were the groups similar at baseline or adjusted for confounder**  **? (2)** | **Was the allocation adequately concealed? (3)** | **Are the animals randomly housed during the experiment? (4)** | **Were the caregivers/ investigators adequately blinded during the course of the experiment? (5)** | **Were animals selected at random during outcome assessment? (6)** | **Was the outcome assessor adequately blinded? (7)** | **Were incomplete outcome data ade- quately addressed? (8)** | **Is the study free of selective outcome reporting? (9)** | **Was the study apparently free of other problems that could cause a high risk of bias? (10)** |
| --- | --- | --- | --- | --- | --- | --- | --- | --- | --- | --- |
| Li et al. 2021 (2) | Unclear | Unclear | Unclear | Unclear | Unclear | Unclear | Unclear | No | Yes | Unclear |
| Liu et al. 2016 (3) | No | Unclear | Unclear | Unclear | Unclear | Unclear | Unclear | No | Yes | Unclear |
| Xie et al. 2017 (4) | No | Unclear | Unclear | Unclear | Unclear | Unclear | Unclear | Yes | Yes | Unclear |
| Wang et al. 2018 (5) | Unclear | Unclear | Unclear | Unclear | Unclear | Unclear | Unclear | Yes | Yes | Unclear |
| Li et al. 2021 (6) | No | Unclear | Unclear | Unclear | Unclear | Unclear | Unclear | Yes | Yes | Unclear |
| Zhang et al. 2020 (7) | Unclear | Unclear | Unclear | Unclear | Unclear | Unclear | Unclear | Yes | Yes | Unclear |
| Wang et al. 2020 (8) | Unclear | Unclear | No | No | Unclear | Unclear | Unclear | Unclear | No | Unclear |
| Yang et al. 2016 (9) | No | Unclear | No | No | Unclear | Unclear | Unclear | Yes | Yes | Unclear |
| Lin et al. 2017 (10) | No | Unclear | No | No | Unclear | Unclear | Unclear | Yes | No | Unclear |

References

(1) Hooijmans CR, Rovers MM, de Vries RBM, Leenaars M, Ritskes-Hoitinga M, Langendam MW. SYRCLE’s risk of bias tool for animal studies*. BMC medical research methodology* (2014) **14**:43. doi: 10.1186/1471-2288-14-43.

(2) Li A, Li Y, Zhang X, Zhang C, Li T, Zhang J, et al. The human milk oligosaccharide 2′-fucosyllactose attenuates β-lactoglobulin–induced food allergy through the miR-146a–mediated toll-like receptor 4/nuclear factor-κB signaling pathway*. Journal of Dairy Science* (2021) **104**:10473. doi: 10.3168/jds.2021-20257.

(3) Liu Z, Yang G, Geng X, Liu J, Mo L, Liu Z, et al. Micro RNA-17-92 cluster mediates interleukin-4-suppressed IL-10 expression in B cells*. American journal of translational research* (2016) **8**:2317–2324.

(4) Xie R, Xu L, Yang L, Wang S, Liu Q, Liu Z, et al. Galectin-1 inhibits oral-intestinal allergy syndrome*. Oncotarget* (2017) **8**:13214–13222. doi: 10.18632/oncotarget.14571.

(5) Wang J, Li S, Li A, Zhang Q, Ni W, Li M, et al. Effect of Lactobacillus acidophilus KLDS 1.0738 on miRNA expression in in vitro and in vivo models of β-lactoglobulin allergy*. Bioscience, Biotechnology, and Biochemistry* (2018) **82**:1955. doi: 10.1080/09168451.2018.1495551.

(6) Li A, Yang J, Zhang C, Chi H, Zhang C, Li T, et al. Lactobacillus acidophilus KLDS 1.0738 inhibits TLR4/NF‐κB inflammatory pathway in β‐lactoglobulin‐induced macrophages via modulating miR‐146a*. J Food Biochem* (2021) **45**. doi: 10.1111/jfbc.13662.

(7) Zhang Q, Ni W, Li Y, Zhang X, Hou J, Meng XC, et al. Analysis of altered miRNA profiling in the colon of a mouse model with β-lactoglobulin allergy*. Allergologia et Immunopathologia* (2020) **48**:666. doi: 10.1016/j.aller.2020.05.007.

(8) Wang J, Zhang Y, Li H, Chen G, Zou Y, Rin K. Immune effects of miRNA and Th17 cells on β-Lg allergy in dietary milk based on mouse model*. Saudi Journal of Biological Sciences* (2020) **27**:3442. doi: 10.1016/j.sjbs.2020.08.028.

(9) Yang L, Li X, Qiu S, Zeng L, Li L, Feng B, et al. Micro RNA-19a suppresses thrombospondin-1 in CD35 + B cells in the intestine of mice with food allergy*. American journal of translational research* (2016) **8**:5503–5511.

(10) Lin R, Liu J, Lu H, Chen Y, Guan L, Liu Z, et al. Micro RNA-155 plays a critical role in the initiation of food allergen-related inflammation in the intestine*. Oncotarget* (2017) **8**:67497–67505. doi: 10.18632/oncotarget.18723.
