# Supplementary material for: MicroRNA expression and their molecular targets in food allergies: a systematic review
Source: Front Immunol. 2025 May 12;16:1524392. doi: 10.3389/fimmu.2025.1524392 (PMC12104090; doi:10.3389/fimmu.2025.1524392)
Supplement: Supplementary file 2 [file Table2.docx]

Supplementary Material

Supplementary Table 2. Risk of bias evaluation of the nonrandomized clinical trials with Newcastle-Ottawa Scale (NOS).

| **Ref** | **Selection** | | | | **Comparability** | **Exposure** | | | **Total Score** |
| --- | --- | --- | --- | --- | --- | --- | --- | --- | --- |
|  | **Is the case definition adequate** | **Representativeness of the cases** | **Selection of controls** | **Definition of controls** | **Comparability of cases and controls on the basis of the design and analysis** | **Ascertainment of Exposure** | **Same method for ascertainment for cases and controls** | **Non-response**  **rate** |  |
| Clemente et al. 2019 (1) | ★ | ★ | / | ★ | ★★ | ★ | ★ | / | 7 |
| Larsen et al. 2018 (2) | ★ | ★ | ★ | ★ | / | ★ | ★ | / | 6 |
| Francuzik et al. 2022 (3) | ★ | ★ | / | ★ | ★ | ★ | / | / | 5 |
| Worm et al. 2022 (4) | ★ | ★ | ★ | ★ | / | / | / | / | 4 |
| Nuñez-Borque et al. 2021 (5) | ★ | ★ | ★ | ★ | ★ | / | ★ | / | 6 |
| Nuñez-Borque et al. 2023 (6) | ★ | ★ | ★ | ★ | ★ | / | ★ | / | 6 |
| D'Argenio et al. 2017 (7) | ★ | ★ | / | ★ | / | ★ | / | / | 4 |

Note: ★= Yes, /= No.

References

(1) Clemente E, Efthymakis K, Carletti E, Capone V, Sperduti S, Bologna G, et al. An explorative study identifies miRNA signatures for the diagnosis of non-celiac wheat sensitivity*. PLoS ONE* (2019) **14**. doi: 10.1371/journal.pone.0226478.

(2) Larsen LF, Juel‐berg N, Hansen A, Hansen KS, Mills ENC, Van Ree R, et al. No difference in human mast cells derived from peanut allergic versus non‐allergic subjects*. Immunity Inflam &amp; Disease* (2018) **6**:416. doi: 10.1002/iid3.226.

(3) Francuzik W, Pažur K, Dalke M, Dölle-Bierke S, Babina M, Worm M. Conflict of interest: MW discloses consulting fees and honoraria from Viatris, Aimmne, and ALK and advisory board membership in DBV-Technologies. MB discloses consulting fees and honoraria from Escient Pharmaceuticals and consulting fees from Health Advances (2022). doi: 10.1172/jci.

(4) Worm M, Alexiou A, Höfer V, Birkner T, Jeanrenaud ACSN, Fauchère F, et al. An interdisciplinary approach to characterize peanut‐allergic patients—First data from the FOOD@ consortium*. Clinical &amp; Translational All* (2022) **12**. doi: 10.1002/clt2.12197.

(5) Nuñez‐borque E, Fernandez‐bravo S, Rodriguez Del Rio P, Alwashali EM, Lopez‐dominguez D, Gutierrez‐blazquez MD, et al. Increased miR‐21‐3p and miR‐487b‐3p serum levels during anaphylactic reaction in food allergic children*. Pediatric Allergy Immunology* (2021) **32**:1296. doi: 10.1111/pai.13518.

(6) Nuñez-Borque E, Fernandez-Bravo S, Rodríguez Del Rio P, Palacio-García L, Di Giannatale A, Di Paolo V, et al. Novel mediator in anaphylaxis: decreased levels of miR-375-3p in serum and within extracellular vesicles of patients*. Front Immunol* (2023) **14**. doi: 10.3389/fimmu.2023.1209874.

(7) D'Argenio V, Del Monaco V, Paparo L, De Palma FDE, Nocerino R, D'Alessio F, et al. Altered miR‐193a‐5p expression in children with cow's milk allergy*. Allergy (Copenhagen)* (2018) **73**:379–386. doi: 10.1111/all.13299.
