# Supplementary material for: MicroRNA expression and their molecular targets in food allergies: a systematic review
Source: Front Immunol. 2025 May 12;16:1524392. doi: 10.3389/fimmu.2025.1524392 (PMC12104090; doi:10.3389/fimmu.2025.1524392)
Supplement: Supplementary file 3 [file Table3.docx]

Supplementary Material

Supplementary Table 3. Risk of bias evaluation of the completely randomized clinical trial with Cochrane Risk of Bias 2 (ROB 2) method (1).

| **Ref** | **Randomization process (D1)** | **Deviations from the intended intervention(D2)** | **Missing outcome data (D3)** | **Measurement of the outcome (D4)** | **Selection of the reported result (D5)** | **Overall** |
| --- | --- | --- | --- | --- | --- | --- |
| Paparo et al. 2019 (2) | Low risk   | High risk   | Low risk   | High risk   | Low risk   | High risk   |

Note: = Low risk of bias, = Some concern, = High risk.

References

(1) Sterne JAC, Savović J, Page MJ, Elbers RG, Blencowe NS, Boutron I, et al. RoB 2: a revised tool for assessing risk of bias in randomised trials*. BMJ* (2019). doi: 10.1136/bmj.l4898.

(2) Paparo L, Nocerino R, Bruno C, Di Scala C, Cosenza L, Bedogni G, et al. Randomized controlled trial on the influence of dietary intervention on epigenetic mechanisms in children with cow’s milk allergy: the EPICMA study*. Sci Rep* (2019) **9**. doi: 10.1038/s41598-019-38738-w.
